# CD155 as a prognostic and predictive biomarker in acute myeloid leukemia

**DOI:** 10.1007/s12672-025-02665-2

**Published:** 2025-05-24

**Authors:** Ashraf Zeidan Abdalla, Salah Mabrouk Khallaf, Asmaa Mohammed Zahran, Nehal Adel Rayan, Ahmed Refaat Abd Elzaher

**Affiliations:** 1 Medical Oncology Department, South Egypt Cancer Institute, Assiut University, Assiut, 71515 Egypt; 2 Clinical Pathology Department, South Egypt Cancer Institute, Assiut University, Assiut, 71515 Egypt

**Keywords:** Acute Myeloid Leukemia, CD155, Survival

## Abstract

**Background:**

Acute myeloid leukemia (AML) is a heterogeneous hematological malignancy with different molecular and genetic alterations. CD155 is expressed on myeloid cells and serves as a recognition molecule for natural killer (NK) cells to induce cytotoxicity. This study aimed to assess the prognostic and predictive value of CD155 in patients with AML.

**Methods:**

We studied the expression of CD155 via flow cytometry in 93 AML patients at initial diagnosis at the Medical Oncology Department, South Egypt Cancer Institute (SECI), Egypt.

**Results:**

Flow cytometry revealed that a mean fluorescence intensity ratio (MFIR) ≥2.68 was associated with high CD155 expression. This criterion was met in 62 patients (66.7%). High expression was associated with a worse composite complete remission (CRc) rate than low expression (29.0 vs. 54.8%; *P* = 0.015). High CD155 expression had significantly shorter relapse-free survival (RFS), with a median of 1.68 (95% CI 0.92–2.43) months (*P* = 0.015), and overall survival (OS), with a median of 1.68 (95% CI 0.74–2.88) months (*P* = 0.041).

**Conclusions:**

High expression of CD155 in adult patients with AML is associated with a lower CRc, shorter RFS, and OS. It could be included within the predictive and prognostic panels.

## Introduction

Acute myeloid leukemia (AML) still represents a significant clinical challenge, as its treatment is hindered by high morbidity and mortality rates largely attributable to chemotherapy resistance and frequent relapses, highlighting a critical need for novel therapeutic approaches [1].

Acute myeloid leukemia (AML) cells evade the immune system through multiple mechanisms, one of which is the upregulation of inhibitory ligands. These cells exhibit increased expression of ligands associated with T-cell regulatory checkpoints, such as cytotoxic T-lymphocyte antigen 4 (CTLA-4), and programmed cell death protein 1 (PD-1). This elevated expression is linked to poorer clinical outcomes in AML [2].

The poliovirus receptor (PVR, CD155), a member of the immunoglobulin (Ig) superfamily, plays a key role in regulating various biological processes, including cell growth, invasion, migration, and immune response modulation, particularly in the context of malignancy development [3]. CD155 is minimally expressed in several normal human tissues, including immune, epithelial, and endothelial cells. However, it is often upregulated in human malignancies, with its heightened activity closely associated with poor prognosis [4].

CD155 is known for its immunoregulatory role in immune cells, where it suppresses cell proliferation and cytokine production by interacting with T cell immunoreceptors with Ig and ITIM domains (TIGIT) on T cells and natural killer cells. This interaction leads to a reduction in GATA-binding protein 3 and interferon regulatory factor 4. Additionally, CD155 exerts a cytotoxic function through its binding with CD226 [5]. However, the underlying mechanisms through which CD155 influences tumor progression require further exploration [6].

It is also involved in the tumor immune response, where the activities of T and NK cells are commonly suppressed by interactions between checkpoint receptors and ligands expressed in the tumor microenvironment. Among these are the TIGIT and CD96 immune checkpoint receptors competing with DNAM-1 to bind with CD155 to suppress DNAM-1- mediated T- and NK cell activation. These interactions occur with different affinities [5]. TIGIT has the highest affinity for CD155, followed by CD96, which has an intermediate affinity, and DNAM-1 has the lowest affinity for CD155 [6].

These molecules are gaining clinical significance in solid tumors, where elevated levels are linked to poor prognosis [7, 8]. However, limited research exists on their predictive and prognostic relevance in AML patients [9].

This study aims to evaluate the predictive and prognostic significance of CD155 in patients with AML.

## Patients and methods

### Patients

A prospective, one-arm, single-center study was conducted on 93 newly diagnosed AML patients at the Medical Oncology Department, South Egypt Cancer Institute (SECI), Assiut University, Egypt from 1st July 2021 to 31st December 2023. The exclusion criteria for this study included patients less than 18 years of age, patients with relapsed or secondary AML, patients with acute promyelocytic leukemia, AML patients with CNS infiltration, and patients with a history of other malignant diseases.

### Diagnosis of AML patients

The diagnosis of AML was established by medical history, clinical examination, complete blood count, standard bone marrow (BM) morphological examination, immunophenotyping, and cytogenetic and molecular studies. Flow cytometric analysis of BM aspirate samples was performed via a fluorescence-activated cell sorter BD FACSCantoTM II triple-laser flow cytometer (Becton Dickenson, San Diego, California, USA) via an initial panel that included the following: CD3, CD4, CD8, CD5, CD10, CD19, CD45, CD33, CD13, CD34, HLA-DR, CD117, and CD14. The secondary panel included cyto-IgM, cyto-MPO, cyto-CD3, CD64, CD36, CD235a, cyto-CD41 and cyto-CD61. Cytogenetic testing was performed for all patients in this study via conventional cytogenetic studies. Fluorescence in situ hybridization (FISH) was performed for cases with insufficient metaphases. Molecular genetic testing was performed to detect FMS-like tyrosine kinase 3-internal tandem duplication (FLT3-ITD) mutations and nucleophosmin-1 (NPM1) mutations in all patients. Risk stratification of our patients was performed based on cytogenetics only because of the unavailability of all molecular analyses at our locality.

### Evaluation of CD155 expression

The expression of CD155 was evaluated in patients at diagnosis via the use of an allophycocyanin (APC) anti-human CD155 (PVR) antibody (BioLegend, San Diego, USA). For each 100 μL BM sample, 10 μL of CD45 and 10 μL of CD155 were added, the tube was incubated at room temperature in the dark for 15 min, and 3 ml of 1X lysing solution was added. The tube was then centrifuged at 2500 rpm for 1 min, the supernatant was then discarded, the mixture was then washed with 3 ml of phosphate-buffered saline (PBS), the tube was centrifuged at 2500 rpm for 1 min, and the supernatant was then discarded. Then, 0.5 ml of PBS was added to the tube and gently mixed. An isotype-matched negative control sample was used in all cases to assess background fluorescence intensity.

The density of cell surface expression of CD155 was determined via flow cytometric analysis on a FACSCanto™ II Triple Laser Flow Cytometer via BD FACSDiva software (BD Biosciences, San Diego, California, USA).

### Statistical analysis

Data was collected and analyzed via the Statistical Package for the Social Sciences (SPSS) version 26 (IBM, Armonk, New York) and GraphPad Prism (version 10.1.1, GraphPad Software, LLC). The data were analyzed via descriptive statistics. Continuous data are expressed as the mean ± standard deviation (SD) or median (range), whereas nominal data are expressed as the frequency (percentage). Categorical data were analyzed via the chi-square test or Fisher’s exact test. Continuous variables were analyzed via the Mann–Whitney test. The diagnostic performance of the MFI cutoff was assessed via a receiver operating characteristic (ROC) curve. Overall survival (OS) and relapse-free survival (RFS) were analyzed via the Kaplan–Meier method and compared via the log-rank test. A Cox proportional hazards model was used to assess the multivariable relationships between prognostic factors and survival outcomes. All *p* values were two-sided, and statistical significance was set at *p* < 0.05.

The objective response rate (ORR) was defined as the percentage of people who achieved a partial response (PR) or complete response (CR) to the treatment following conventional induction therapy with 3 days of anthracycline and 7 days of cytarabine (“3 + 7” protocol without targeted therapy). Composite complete response (CRc): CR + CR with incomplete platelet recovery (CRp) + CR with incomplete neutrophil recovery (CRi) using modified International Working Group (IWG) criteria [10].

Overall survival (OS) was defined as the time from the date of diagnosis to the date of death from any cause or the date of last follow-up. RFS was defined as the interval between the date of complete remission and the date of any of the following events: relapse, death, or last follow-up; whichever occurred first.

## Results

The current study was conducted on 93 AML patients. The median age was 38 (18–66 years), with a slight male predominance (53.8%). Anemia was the most common clinical presentation (36.6%), followed by fever and bleeding tendency with incidences of 21.5 and 17.2%, respectively. The median blast percentages in PB and BM were 45% (0–95%) and 70% (20–99%), respectively. According to the World Health Organization (WHO) 2022 classification, acute myelomonocytic leukemia and acute monocytic leukemia represented the most frequent subtypes (63.4%), followed by acute leukemia with maturation (20.4%).

### CD155 median fluorescence intensity (MFI) cutoff

**Fig. 1:**
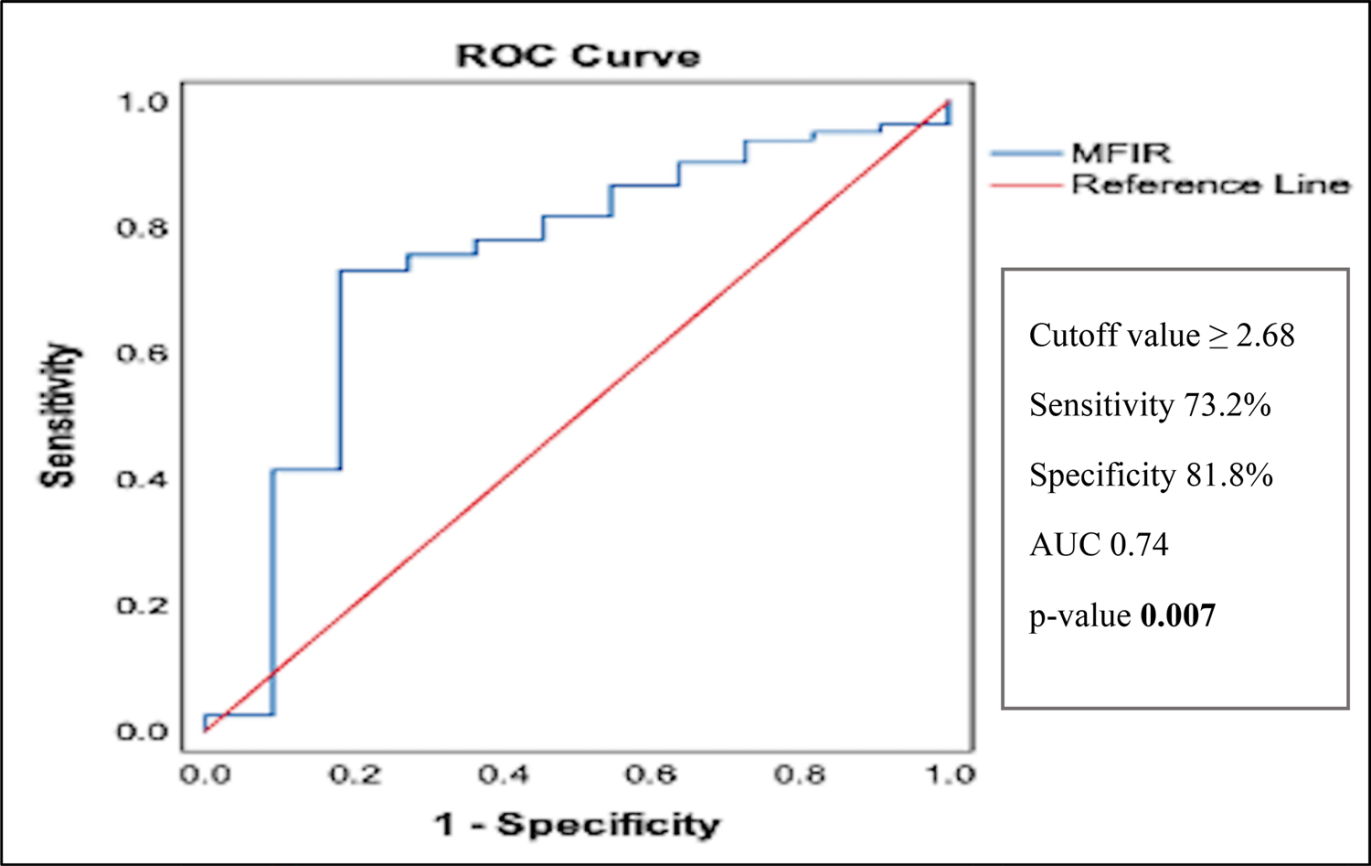
Diagnostic performance of CD155 MFIR

The median fluorescence intensity (MFI) for CD155 and the MFI for the isotype-matched negative control were obtained from histograms. CD155 expression was evaluated by calculating the MFI ratio (MFIR), which was calculated by dividing the MFI of CD155 by the MFI of the isotype-matched negative control. A cutoff for MFIR was calculated via a receiver operating characteristic (ROC) curve according to survival for all patients. At a cutoff value of ≥2.68, 73.2% sensitivity and 81.8% specificity were observed for predicting the survival in the studied patients with an area under the curve of 0.74 and a *p*-value of 0.007 (Fig. 1).

Based on this CD155 cutoff, MFIR < 2.68 was considered a low expression, while MFIR ≥ 2.68 was considered a high expression. Thiry-one patients (33.3%) had low CD155 expression, and 62 patients (66.7%) had high CD155 expression.

There were insignificant differences between patients with high and low CD155 expression in terms of demographic data; baseline laboratory data; and cytogenetic, molecular, and risk stratification data, as summarized in Tables 1 and 2. Patients with high CD155 expression had a worse composite complete response (CRc) rate than those with low CD155 expression (29.0 vs. 54.8%; *p* = 0.015) (Table 3).

**Table 1:** Demographic data of patients’ comparative analysis based on CD155 expression

	Low expression (N = 31)		High expression (N = 62)		*P* value
	No	%	No	%	
Age/years (median (range)) *	40.45 (18–66)		34.5 (18–66)		0.177
Gender					
• Female	16	51.6%	27	43.5%	0.462
• Male	15	48.4%	35	56.5%	
Performance status					
• ECOG-0/1	20	64.5%	29	46.8%	0.106
• ECOG-2	11	35.5%	33	53.2%	
Clinical presentation					
• Fever	5	16.1%	15	24.2%	0.131
• Bleeding	5	16.1%	11	17.7%	
• Hypertrophied gum	0	0.0%	2	3.2%	
• Anemic manifestation	11	35.5%	23	37.1%	
• Organomegaly	7	22.6%	2	3.2%	
• Others	3	9.7%	9	14.6%	
WHO classification 2022					
• AML with minimal differentiation	1	3.2%	4	6.5%	0.967
• AML without maturation	3	9.7%	7	11.3%	
• AML with maturation	7	22.6%	12	19.4%	
• Acute myelomonocytic leukemia	16	51.6%	31	50.0%	
• Acute monocytic leukemia	4	12.9%	8	12.9%	

Laboratory data	Median	Range	Median	Range	
WBC *	28.2	1.3–295.0	28.5	1.3–294.0	0.948
Hb *	7.8	4.6–11.9	8.2	3.9–14.4	0.165
PLTs *	40.0	14.0–524.0	37.5	6.0–187.0	0.103
PB blast *	40.0	0.0–95.0	45.5	0.0–95.0	0.602
BM blasts *	62.0	20.0–98.0	71.0	23.0–99.0	0.381
LDH *	442.0	133.0–2184.0	525.5	127.0–4208.0	0.131

Chi square test, *Mann-Whitny test, *P* between two groups, **significant (*P* value <0.05)

*DIC* Disseminated intravascular coagulopathy, *ECOG* Eastern Cooperative Oncology Group, *WHO* World Health Organization, *AML* Acute myeloid leukemia, *WBC* White blood cell, *Hb* hemoglobin level, *PLTs* platelet count, *BM* Bone marrow, *PB* Peripheral blood, *LDH* Lactate dehydrogenase

**Table 2:** Cytogenetic and molecular data in patients’ comparative analysis based on CD155 expression

	Low expression (N = 31)		High expression (N = 62)		*P* value
	No	%	No	%	
Cytogenetics					
• Normal Karyotype	17	54.8%	39	62.9%	0.320
• t(8;21)	4	12.9%	5	8.1%	
• inv(16)/t(16;16)	4	12.9%	7	11.3%	
• MLL rearrangement	3	9.7%	3	4.8%	
• MECOM rearrangement	1	3.2%	1	1.6%	
• t(9;22)	2	6.5%	0	0.0%	
• Trisomy 8	0	0.0%	4	6.5%	
• Monosomy 7	0	0.0%	1	1.6%	
• Complex karyotype	0	0.0%	2	3.2%	
Molecular					
FLT3-ITD					0.879
• Wild type	20	64.5%	39	62.9%	
• Mutated	11	35.5%	23	37.1%	
NPM1					0.850
• Mutated	11	35.5%	11	17.7%	
• Not mutated	20	64.5%	51	82.3%	
Cytogenetic risk stratification					0.361
• Favorable	8	25.8%	12	19.4%	
• Intermediate	17	54.8%	43	69.3%	
• Unfavorable	6	19.4%	7	11.3%	

Chi-Square test, *P* between two groups, significant (*P* value <0.05)

*t* Translocation, *inv* Inversion, *FLT3-ITD* FMS-like tyrosine kinase 3/internal tandem duplication, *NPM1* Nucleophosmin 1

**Table 3:** Association between CD155 & response to induction therapy

Response	Low expression (N = 31)		High expression (N = 62)		*P* value
	No	%	No	%	
ELN 2022 response criteria					
• CR-MRD	1	3.2%	0	0.0%	0.127
• CR	15	48.4%	18	29.0%	
• Incomplete CR	1	3.2%	0	0.0%	
• Partial response	2	6.5%	7	11.3%	
• Primary refractory	4	12.9%	15	24.2%	
• Death from any cause	8	25.8%	22	35.5%	
Composite Complete Response					
• Yes	17	54.8%	18	29.0%	0.015
• No	14	45.2%	44	71.0%	
Partial response*					
• Yes	2	6.5%	7	11.3%	0.713
• No	29	93.5%	55	88.7%	
Objective response rate (CR, CR-MRD, and PR)					
• Yes	19	61.3%	25	40.3%	0.056
• No	12	38.7%	37	59.7%	
Treatment failure (primary refractory and death from any cause)					
• Yes	12	38.7%	37	59.7%	0.056
• No	19	61.3%	25	40.3%	

Chi-Square test, *Fisher’s Exact Test, *P* between two groups, significant (*P* value <0.05)

**Fig. 2:**
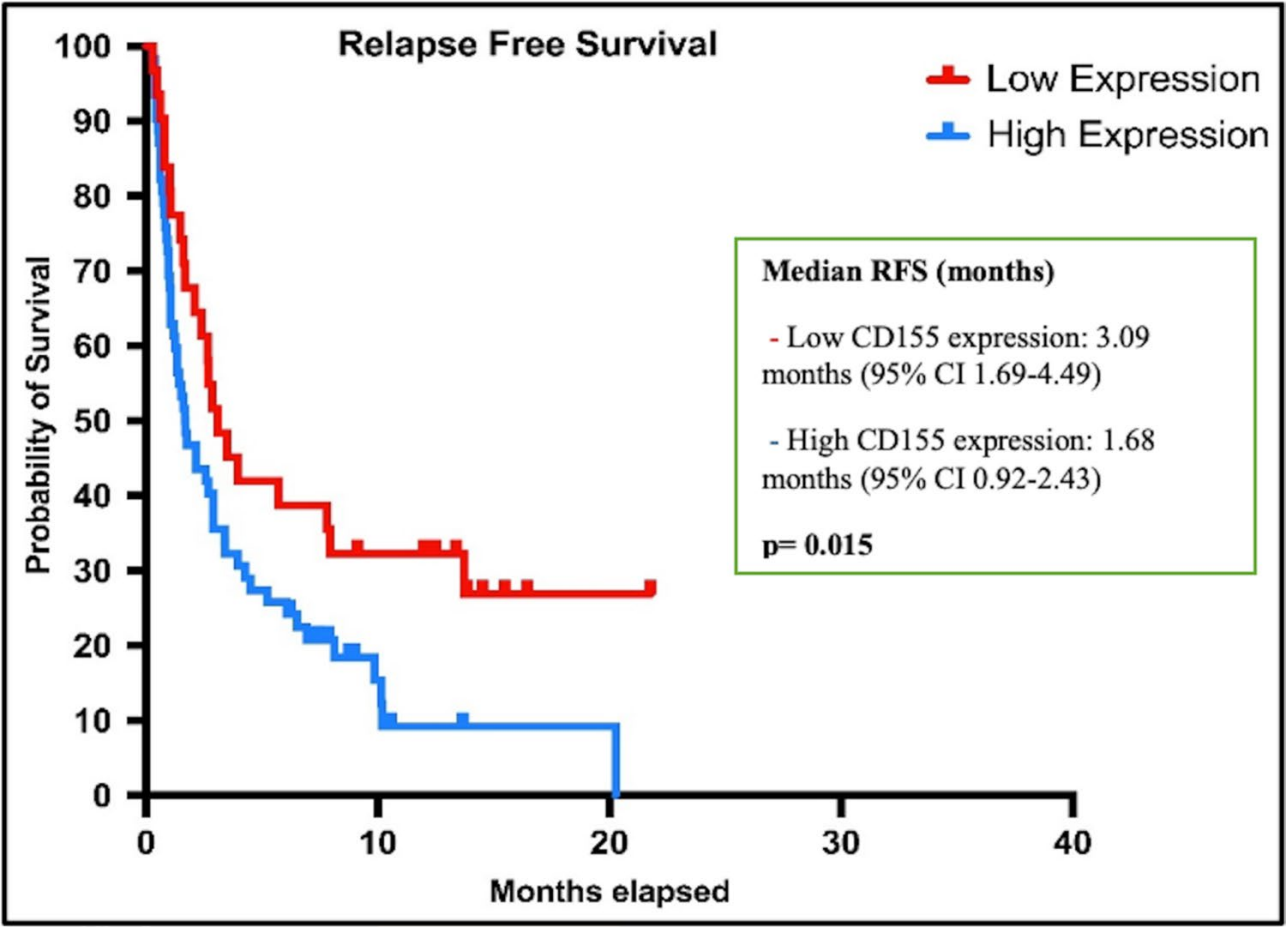
Kaplan Meier curves showing relapse-free survival according to CD155 expression status

**Fig. 3:**
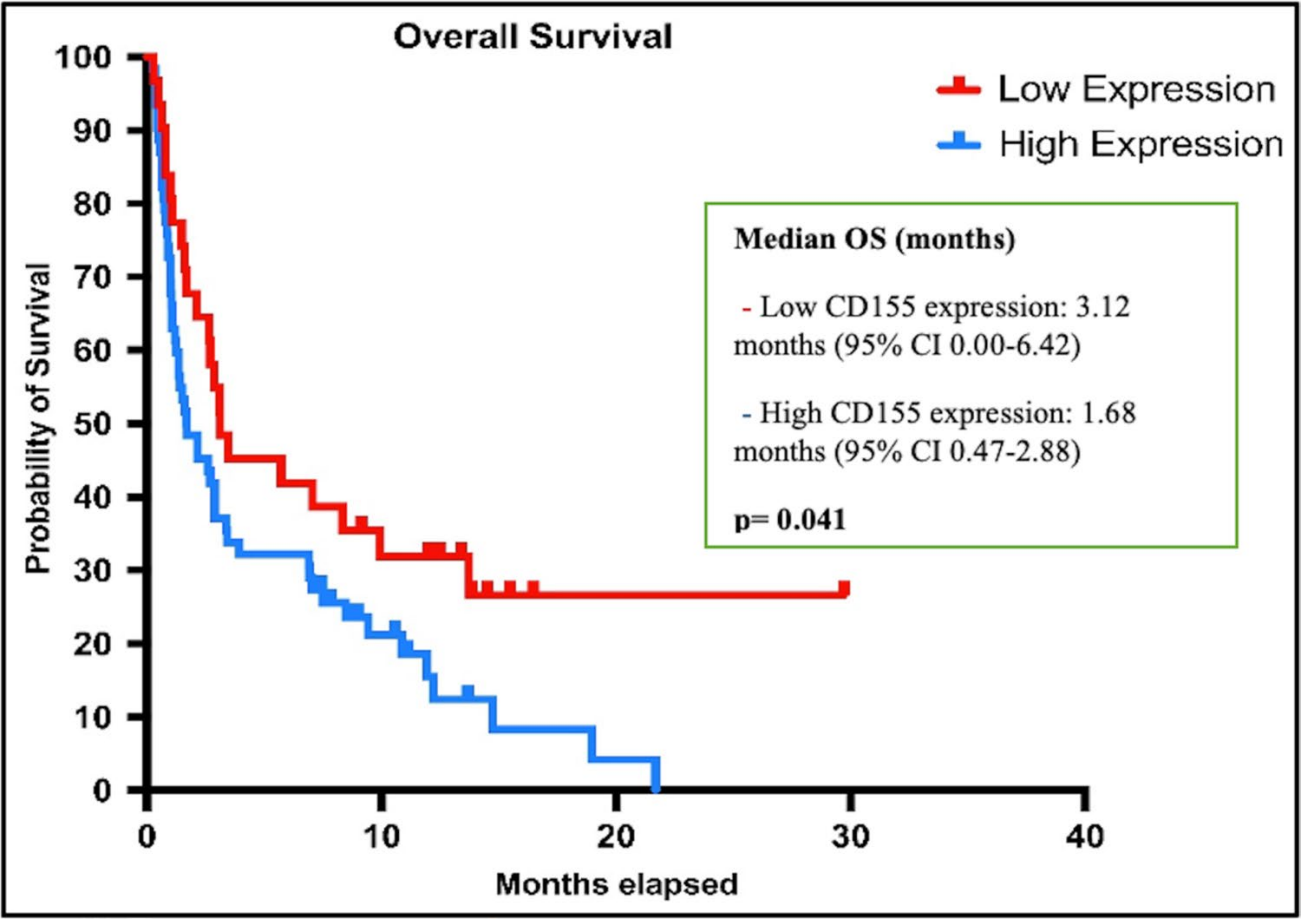
Kaplan Meier curves showing overall survival according to CD155 expression status

Patients with high CD155 expression had significantly shorter RFS than those with low CD155 expression, with a median of 1.68 vs. 3.09 months (95% CI 0.92–2.43) (*p* = 0.015), and OS was significantly shorter in patients with high CD155 expression than in patients with low CD155 expression with a median of 1.68 vs. 3.12 months 95% CI 0.47–2.88) (*p* = 0.041) (Figs. 2 and 3).

COX regression analysis was used to predict survival, and univariate analysis revealed a significant association between OS and the following variables: age (*p* < 0.000), PS (*p* = 0.012), and CD155 expression (*p* = 0.044). However, there was no association between OS and the other studied factors. Multivariate analysis revealed that age and C155 were independent prognostic risk factors, with *p*-values of 0.000 and 0.011, respectively (Table 4).

**Table 4:** Cox Proportional Hazard Regression analysis for OS for CD155 expression and patient’s Characteristic (N = 93)

Parameter	Univariate analysis				Multivariate analysis			
	HR	95% CI		*P*	HR	95% CI		*p*
Age	1.04	1.02	1.06	0.000	1.05	1.02	1.07	0.000
Gender	0.74	0.47	1.16	0.192				
PS	1.79	1.14	2.82	0.012	1.22	0.74	1.99	0.439
WBC	1.00	0.99	1.01	0.470				
Hb	1.02	0.92	1.13	0.746				
RBCs	0.89	0.69	1.14	0.359				
PLT	0.99	0.99	1.00	0.087				
PB	1.00	0.99	1.01	0.701				
BM blast	1.01	0.99	1.02	0.344				
LDH	1.00	1.00	1.00	0.599				
WHO 2022 classification								
Acute leukemia without maturation	0.98	0.33	2.97	0.982				
Acute leukemia with maturation	0.52	0.19	1.44	0.207				
Acute myelomonocytic leukemia	0.54	0.21	1.38	0.202				
Acute monocytic leukemia	0.54	0.17	1.66	0.282				
Cytogenetics abnormality	0.77	0.48	1.25	0.291				
FLT3	1.25	0.78	1.98	0.357				
NPM1	1.09	0.63	1.87	0.755				
Cytogenetic risk stratification								
Favorable	–	–	–	–				
Intermediate	1.29	0.73	2.26	0.382				
Unfavorable	1.04	0.48	2.28	0.921				
CD 155	1.67	1.01	2.75	0.044	2.00	1.17	3.43	0.011

ox regression was used

*PS* Performance Status, *WBC* White blood cell, *Hb* Hemoglobin level, *RBCs* Red blood cells, *PLTs* Platelet count, *BM* Bone marrow, *PB* Peripheral blood, *LDH* Lactate dehydrogenase, *WHO* World Health Organization, *FLT3-ITD* FMS-like tyrosine kinase 3/internal tandem duplication, *NPM1* Nucleophosmin 1, *HR* Hazard’s Ratio, *CI* Confidence Interval

Logistic regression analysis was used for the prediction of response, and univariate analysis revealed a significant association between response and the following variables: age (*p* = 0.027), PS (*p* = 0.012), platelet count (*p* = 0.028), blast count BM (*p* = 0.053), and CD155 expression (*p* = 0.017). However, there was no association between response and the other studied factors. Multivariate analysis revealed significant association with age (*p* = 0.010) and CD155 expression (*p* = 0.023) which are independent risk factors for the response to chemotherapy (Table 5).

**Table 5:** Logistic regression analysis for prediction of response within AML cases (N = 93)

Parameter	Univariate analysis				Multivariate analysis			
	OR	95% CI		*P*	OR	95% CI		*p*
Age	0.96	0.92	0.99	0.027	0.95	0.91	0.98	0.010
Gender	1.50	0.64	3.51	0.350				
PS	0.75	0.32	1.75	0.504				
WBC	1.00	0.99	1.01	0.719				
Hb	0.97	0.79	1.19	0.784				
RBCs	1.15	0.71	1.87	0.568				
PLT	1.01	1.00	1.02	0.028	1.01	0.99	1.02	0.063
PB	0.99	0.98	1.01	0.432				
BM blast	0.98	0.96	1.00	0.053	0.98	0.96	1.01	0.164
LDH	1.00	1.00	1.01	0.516				
WHO 2022 classification								
Acute leukemia without maturation	2.25	0.11	45.72	0.598				
Acute leukemia with maturation	0.34	0.03	3.68	0.378				
Acute myelomonocytic leukemia	0.26	0.03	2.51	0.245				
Acute monocytic leukemia	0.25	0.02	2.95	0.271				
Cytogenetics abnormality	2.09	0.88	4.98	0.093				
FLT3-ITD	0.57	0.23	1.39	0.216				
NPM1	1.39	0.51	3.85	0.520				
Cytogenetic risk stratification								
Favorable	–	–	–	–				
Intermediate	1.96	0.47	8.11	0.356				
Adverse	0.74	0.21	2.57	0.637				
CD 155	0.34	0.14	0.83	0.017	0.30	0.11	0.85	0.023

Logistic regression was used

*PS* performance status, *WBC* White blood cell, *Hb* hemoglobin level, *RBCs* red blood cells, *PLTs* platelet count, *BM* Bone marrow, *PB* Peripheral blood, *LDH* Lactate dehydrogenase, *WHO* World Health Organization, *FLT3-ITD* FMS-like tyrosine kinase 3/internal tandem duplication, *NPM1* Nucleophosmin 1, *OR* Odd’s Ratio, *CI* Confidence Interval

## Discussion

AML is a hematologic malignancy defined by the clonal proliferation of immature hematopoietic precursors, which infiltrate the bone marrow and disrupt normal hematopoiesis. It remains a significant clinical challenge, associated with high morbidity and mortality rates [11].

CD155 plays a key immunoregulatory role and exhibits dual functions in tumor immunity. The equilibrium between CD155/CD226 (stimulatory interactions) and CD155/TIGIT or CD155/CD96 (inhibitory interactions) is essential for maintaining normal NK and T-cell activity. However, this balance can be disrupted within the tumor microenvironment, where inhibitory signaling predominates due to decreased CD226 and increased TIGIT expression. Additionally, tumor cells often overexpress CD155, a phenomenon associated with poor prognosis [12].

The expression of CD155 among AML patients is heterogenous and few studies on its prognostic value in AML patients and its clinical significance in the stratification of those patients is still unclear [9].

The current study revealed insignificant differences between patients with high and low CD155 expression in terms of demographic data; baseline laboratory data; cytogenetic and molecular data, and cytogenetic risk stratification at the time of diagnosis.

Our results are close to those of the Egyptian study performed by Mohamed et al. [13], where the relationships between the CD155 level and patient sex, hemoglobin concentration, total leukocytic count, platelet count, and BM blast percentage at the time of diagnosis were statistically insignificant.

In contrast to our results, Stammand et al. [14] revealed a clear positive association between increasing leukemic cell numbers and CD155 plasma levels. Additionally, they reported a greater CD155 level in patients who had unfavorable cytogenetic risk. This discrepancy may be explained by the limited number of patients in our study.

The results from this study revealed that patients with low CD155 expression had significantly longer OS and RFS than did those with high CD155 expression, which was consistent with the results reported by Stamm et al. [14]. These findings are in agreement with other published data by Klingler et al. [15], who reported that high CD155 expression was associated with poor OS.

Our analysis of patient cohorts revealed that CD155 expression was an independent risk factor for survival. In support of our findings regarding the clinical significance of CD155 expression in AML, in patients with pancreatic cancer, high CD155 expression also represents an independent prognostic factor for OS [16]. Similar to our results, Nadia et al. [17]; revealed via multivariate analysis; that only CD155 was significantly associated with shorter overall survival in AML patients.

To the best of our knowledge, no previous data is using CD155 as a predictive factor for induction chemotherapy, however, our data confirms the valuable role of CD155 to be an independent good predictive factor, and it needs further studies for validation.

The limitation of this study is a relatively small sample size, the reason is the rarity of this disease in our locality. The other limitation is the unavailability of targeted therapy due to financial issues.

## Conclusion

Our study demonstrated that elevated CD155 expression is linked to a lower response rate, as well as reduced relapse-free and overall survival. Cox regression analysis identified CD155 as an independent risk factor for overall survival. Its assessment could be incorporated into routine laboratory practice for risk stratification, aiding in the classification of patients into low- and high-risk groups who may require additional therapeutic strategies, including novel immunotherapies. In the future, CD155 could serve as a potential therapeutic target, like PD-L1 and CTLA-4, offering new treatment opportunities for these patients.

## Data Availability

The data that support the findings of this study are not openly available due to reasons of sensitivity and are available from the corresponding author upon reasonable request.

## Abbreviations

AML: Acute myeloid leukemia
APC: Allophycocyanin
BM: Bone marrow
CR: Complete response
CRc: Composite complete response
Cri: CR with incomplete neutrophil recovery
CRp: CR with incomplete platelet recovery
FISH: Fluorescence in situ hybridization
FLT3-ITD: FMS-like tyrosine kinase 3/internal tandem duplication
Ig: Immunoglobulin
IWG: International Working Group
MFI: Median fluorescence intensity
MFIR: Mean fluorescence intensity ratio
NK: Natural killer
NPM1: Nucleophosmin 1
ORR: Objective response rate
OS: Overall survival
PBS: Phosphate buffered saline
PVR: Poliovirus receptor
RFS: Relapse free survival
ROC: Receiver Operating Characteristic
SD: Standard deviation
SECI: South Egypt Cancer Institute
SPSS: Statistical Package for The Social Science
TIGIT: T cell immunoreceptor with Ig and ITIM domains
WHO: World Health Organization
